# Supplementary material for: Synthetic data generation for a longitudinal cohort study – evaluation, method extension and reproduction of published data analysis results
Source: Sci Rep. 2024 Jun 22;14:14412. doi: 10.1038/s41598-024-62102-2 (PMC11193715; doi:10.1038/s41598-024-62102-2)
Supplement: Supplementary file 1 — Supplementary Information. [file 41598_2024_62102_MOESM1_ESM.pdf]

# Supplementary Information

## DONALD Data

In Table S1, metadata for the DONALD dataset can be seen that are described in the following. Each participant gets a personal number – a randomly generated unique identifier. In addition, each participating family gets a randomly generated family number. A family consists of the mother, the father, the biological, the adoptive, and the foster children. The sex of each participant is noted as either male or female. For every three-day weighed dietary record, the age of the participant is noted together with the date of the dietary record. Thereby, a specific time variable is created for time trend analyses, where the first included record in this evaluation was considered the baseline time, i.e., time = 0. Therefore, time ranged between 0 and 31 years.

Most variables describe the nutrient intake as a percentage of the total energy intake per day (%E/d). This total energy intake is also given, in kilocalories per day (kcal/d).

In addition, a numerical value for the basal metabolic rate (BMR) is reported, which is the amount of calories that are burnt during rest [3]. Another variable introduced is the boolean variable of underreporting. Thereby, the plausibility of the reported values is determined based on a comparison to standard value ranges according to Sichert-Heilert *et al.* [4]. Two further measures are reported for each visit concerning the weight of the participant. The first one is the determination of obesity according to Cole *et al.* [1] and the other one is the well-known body mass index (BMI). Finally, socioeconomic factors are taken into account, consisting of the BMI of the mother of the participant, whether she is currently employed, and whether she completed her A-level (in German *Fachabitur* or *Abitur* - corresponding to 12 years of school education). For every three-day weighed dietary record, the number of weekdays or weekend days was recorded as well (*wo_tage*).

## VAMBN Settings

Table S2 summarises the different module selections that need to be decided before the use of VAMBN. Our black- and whitelists, which are needed for the Bayesian Network learning, contain the basic constraints that an edge can never go back in time, and that the standalone variables cannot depend on any other variable. Moreover, the used hyperparameters for training can be found in Table S3.

## Real-world Analyses

Table S4 contains the raw values of the trend analyses for both age and time trends for the two different module settings ((i) and (ii)).

Table S1: Overview of the variables contained within the DONALD dataset

| Variable Description | Variable name | Variable type | Unit |
|---|---|---|---|
| Personal number | pers_ID | numerical | - |
| Family number | fam_ID | numerical | - |
| Gender | sex | categorical | - |
| Age on first day of protocol | age | numerical | years |
| Time variable | time | numerical | years |
| Daily energy intake | e_cal | numerical | kcal/d |
| Protein intake | EW_p | numerical | %E/d |
| Fat intake | Fett_p | numerical | %E/d |
| Carbohydrate intake | KH_p | numerical | %E/d |
| Glucose intake | Gluc_p | numerical | %E/d |
| Fructose intake | Fruc_p | numerical | %E/d |
| Galactose intake | Galac_p | numerical | %E/d |
| Monosaccharide intake | MSacch_p | numerical | %E/d |
| Saccharose intake | Sacch_p | numerical | %E/d |
| Maltose intake | MALT_p | numerical | %E/d |
| Lactose intake | LACT_p | numerical | %E/d |
| Disaccharide intake | DISACCH_p | numerical | %E/d |
| Total sugar intake | ZUCK_p | numerical | %E/d |
| Added sugar | ZUZU_p | numerical | %E/d |
| Free sugar | free_s_p | numerical | %E/d |
| Free sugar from juice | fs_saft_p | numerical | %E/d |
| Free sugar from fruits and vegetables | fs_obge_p | numerical | %E/d |
| Free sugar from sugar and sweets | fs_sp_p | numerical | %E/d |
| Free sugar from bread and cake | fs_bc_p | numerical | %E/d |
| Free sugar from other sources | fs_oth_p | numerical | %E/d |
| Free sugar from dairy products | fs_dai_p | numerical | %E/d |
| Free sugar from Sugar Sweetened Beverages (SSB) | fs_ssb_p | numerical | %E/d |
| Number of weekdays | wo_tage | categorical | - |
| Basal metabolic rate (BMR) | bmr | numerical | - |
| Underreporting | underrep | categorical | - |
| Overweight status | ovw | categorical | - |
| Body Mass Index (BMI) | bmi | numerical | kg/m2 |
| Overweight status of the mother | m_ovw | categorical | - |
| Current employment of the mother | m_employ | categorical | - |
| High maternal educational status of the mother | m_schulab | categorical | - |

Table S2: Module selections for different VAMBN-based models*.

| Module Setting | Time | Nutrition | Socioeconomic Factors | Anthropometric Data |
|---|---|---|---|---|
| (i) | age, time | e_cal, EW_p, Fett_p, KH_p, Gluc_p, Fruc_p, Galac_p, MSacch_p, Sacch_p, MALT_p, LACT_p, DISACCH_p, ZUCK_p, ZUZU_p, free_s_p, fs_saft_p, fs_obge_p, fs_sp_p, fs_bc_p, fs_oth_p, fs_dai_p, fs_ssb_p, wo_tage | bmr, underrep, ovw, bmi | m_ovw, m_employ, m_schulab |
| (ii) | age, time, ZUZU_p | e_cal, EW_p, Fett_p, KH_p, Gluc_p, Fruc_p, Galac_p, MSacch_p, Sacch_p, MALT_p, LACT_p, DISACCH_p, ZUCK_p, free_s_p, fs_saft_p, fs_obge_p, fs_sp_p, fs_bc_p, fs_oth_p, fs_dai_p, fs_ssb_p, wo_tage | bmr, underrep, ovw, bmi | m_ovw, m_employ, m_schulab |
| (iii) | age, time, ZUCK_p | e_cal, EW_p, Fett_p, KH_p, Gluc_p, Fruc_p, Galac_p, MSacch_p, Sacch_p, MALT_p, LACT_p, DISACCH_p, ZUZU_p, free_s_p, fs_saft_p, fs_obge_p, fs_sp_p, fs_bc_p, fs_oth_p, fs_dai_p, fs_ssb_p, wo_tage | bmr, underrep, ovw, bmi | m_ovw, m_employ, m_schulab |

*Note that fam_ID and sex are standalone variables for each setting.

Table S3: Hyperparameters used for HI-VAE training

| Hyperparameter | Module | Value |
|---|---|---|
| Learning rate | Times<br>Nutrition<br>Anthropometric<br>Socioeconomic | 0.01 |
| Batch Size | Times<br>Nutrition<br>Anthropometric<br>Socioeconomic | 127 |
| Y-Dimensionality | Times<br>Nutrition<br>Anthropometric<br>Socioeconomic | 1 |
| S-Dimensionality | Times<br>Nutrition | 1 |
| | Anthropometric<br>Socioeconomic | 2 |
| LSTM-Dimensionality | Times<br>Nutrition<br>Anthropometric<br>Socioeconomic | 20 |

Table S4: Results of polynomial mixed-effect models for age and time trends of added sugar intake for the different generated datasets

| Method | $Age$ ($p$) | $Age^2$ ($p$) | $Age^3$ ($p$) | $Time$ ($p$) | $Time^2$ ($p$) | $Time^3$ ($p$) |
|---|---|---|---|---|---|---|
| Original Data* | 1.350827 (<0.0001) | -0.099685 (<0.0001) | 0.002137 (0.0002) | -0.0.348131 (<0.0001) | 0.026152 (<0.0001) | -0.000599 (<0.0001) |
| | 1.430963 (<0.0001) | -0.115705 (<0.0001) | 0.002723 (<0.0001) | 0.025684 (0.223012) | -0.001424 (0.333201) | 2.8e-05 (0.370199) |
| | 1.408156 (<0.0001) | -0.111171 (<0.0001) | 0.002584 (<0.0001) | -0.003557 (0.864749) | 4e-06 (0.998019) | -2e-06 (0.945519) |
| | 1.393149 (<0.0001) | -0.112023 (<0.0001) | 0.002664 (<0.0001) | 0.024316 (0.244942) | -0.002527 (0.083921) | 6e-05 (0.049591) |
| | 1.342718 (<0.0001) | -0.105812 (<0.0001) | 0.002428 (<0.0001) | -0.003396 (0.87088) | 0.000347 (0.812486) | -1.3e-05 (0.683313) |
| | 1.38336 (<0.0001) | -0.108266 (<0.0001) | 0.002462 (<0.0001) | -0.012054 (0.561269) | 0.001444 (0.320452) | -4e-05 (0.18968) |
| | 1.476569 (<0.0001) | -0.118884 (<0.0001) | 0.00281 (<0.0001) | -0.005234 (0.798662) | 7.9e-05 (0.956205) | -4e-06 (0.893813) |
| | 1.417386 (<0.0001) | -0.113722 (<0.0001) | 0.002656 (<0.0001) | 0.004955 (0.812989) | -0.000329 (0.822024) | 1e-05 (0.746472) |
| | 1.440692 (<0.0001) | -0.11426 (<0.0001) | 0.002631 (<0.0001) | 0.014392 (0.479681) | -0.001513 (0.291507) | 3.5e-05 (0.242204) |
| | 1.52464 (<0.0001) | -0.123319 (<0.0001) | 0.002921 (<0.0001) | -0.005849 (0.779298) | 0.000798 (0.582831) | -2.1e-05 (0.491004) |
| | 1.478183 (<0.0001) | -0.121326 (<0.0001) | 0.002939 (<0.0001) | -0.01 (0.623962) | 0.00043 (0.764083) | -7e-06 (0.828089) |
| | 1.416625 (<0.0001) | -0.113158 (<0.0001) | 0.002644 (<0.0001) | -0.009194 (0.65675) | 0.0007 (0.628279) | -1.3e-05 (0.679093) |
| | 1.468699 (<0.0001) | -0.116248 (<0.0001) | 0.002683 (<0.0001) | -0.021168 (0.303979) | 0.000888 (0.540096) | -1.3e-05 (0.666859) |
| | 1.362627 (<0.0001) | -0.110525 (<0.0001) | 0.002614 (<0.0001) | 0.024577 (0.228469) | -0.000575 (0.687768) | -2e-06 (0.958185) |
| | 1.429001 (<0.0001) | -0.113265 (<0.0001) | 0.002622 (<0.0001) | -0.021336 (0.306943) | 0.001751 (0.229002) | -4.4e-05 (0.152824) |
| | 1.421166 (<0.0001) | -0.115259 (<0.0001) | 0.002743 (<0.0001) | 0.021191 (0.303541) | -0.002611 (0.070101) | 6.7e-05 (0.027548) |
| | 1.447328 (<0.0001) | -0.114806 (<0.0001) | 0.002693 (<0.0001) | 0.007654 (0.71308) | -0.001309 (0.368884) | 2.5e-05 (0.411436) |
| | 1.365656 (<0.0001) | -0.106593 (<0.0001) | 0.002449 (<0.0001) | -0.035603 (0.08799) | 0.002134 (0.144344) | -5.1e-05 (0.098494) |
| | 1.399197 (<0.0001) | -0.109818 (<0.0001) | 0.0025 (<0.0001) | -0.01189 (0.565467) | 0.000805 (0.578397) | -2.2e-05 (0.465431) |
| | 1.387137 (<0.0001) | -0.108345 (<0.0001) | 0.002413 (<0.0001) | -0.001731 (0.933152) | 0.000121 (0.933097) | 1e-06 (0.972541) |
| | 1.358621 (<0.0001) | -0.104747 (<0.0001) | 0.002335 (<0.0001) | -0.03694 (0.072523) | 0.001696 (0.238648) | -2.5e-05 (0.403834) |
| | 1.428979 (<0.0001) | -0.113885 (<0.0001) | 0.002644 (<0.0001) | -0.011523 (0.582107) | 0.000795 (0.586429) | -2.1e-05 (0.487305) |
| | 1.4598 (<0.0001) | -0.119574 (<0.0001) | 0.002879 (<0.0001) | -0.005296 (0.798037) | 0.000549 (0.705309) | -9e-06 (0.77996) |
| | 1.386755 (<0.0001) | -0.109129 (<0.0001) | 0.002485 (<0.0001) | -0.002027 (0.921946) | 0.000281 (0.846035) | -1.2e-05 (0.697522) |
| | 1.386846 (<0.0001) | -0.1092 (<0.0001) | 0.002499 (<0.0001) | -0.021206 (0.30399) | 0.001618 (0.261885) | -4.2e-05 (0.170433) |
| VAMBN-MT (i) 10,000 | 1.335938 (<0.0001) | -0.104791 (<0.0001) | 0.00239 (<0.0001) | 0.00188 (0.928299) | -0.00016 (0.913141) | 1e-06 (0.964105) |
| | 1.357753 (<0.0001) | -0.105788 (<0.0001) | 0.002385 (<0.0001) | 0.039295 (0.058432) | -0.003142 (0.030688) | 6.4e-05 (0.036516) |
| | 1.461898 (<0.0001) | -0.114953 (<0.0001) | 0.002655 (<0.0001) | -0.02713 (0.187143) | 0.001143 (0.427771) | -2.4e-05 (0.435993) |
| | 1.407884 (<0.0001) | -0.110518 (<0.0001) | 0.002541 (<0.0001) | 0.020444 (0.322373) | -0.002323 (0.108) | 5.1e-05 (0.094491) |
| | 1.317254 (<0.0001) | -0.102333 (<0.0001) | 0.002291 (<0.0001) | 0.027303 (0.188922) | -0.00276 (0.057939) | 6.4e-05 (0.034668) |
| | 1.345957 (<0.0001) | -0.105435 (<0.0001) | 0.002385 (<0.0001) | 0.008335 (0.685253) | -0.000206 (0.886198) | -1e-06 (0.981259) |
| | 1.293585 (<0.0001) | -0.102001 (<0.0001) | 0.002362 (<0.0001) | -0.031494 (0.122066) | 0.002192 (0.126255) | -5.6e-05 (0.06444) |
| | 1.475885 (<0.0001) | -0.118306 (<0.0001) | 0.002808 (<0.0001) | -0.023357 (0.255611) | 0.001337 (0.353625) | -3e-05 (0.318661) |
| | 1.428994 (<0.0001) | -0.111113 (<0.0001) | 0.002519 (<0.0001) | -0.025981 (0.210783) | 0.001134 (0.433863) | -2.2e-05 (0.473848) |
| | 1.476203 (<0.0001) | -0.120321 (<0.0001) | 0.002882 (<0.0001) | 0.002931 (0.887087) | -0.000183 (0.898464) | 3e-06 (0.922449) |
| | 1.474939 (<0.0001) | -0.121196 (<0.0001) | 0.002974 (<0.0001) | -0.020884 (0.31299) | 0.001194 (0.409684) | -2.7e-05 (0.372887) |
| | 1.418087 (<0.0001) | -0.111836 (<0.0001) | 0.002565 (<0.0001) | -0.021492 (0.298061) | 0.001728 (0.233093) | -4.2e-05 (0.164551) |
| | 1.444998 (<0.0001) | -0.114757 (<0.0001) | 0.002667 (<0.0001) | -0.023323 (0.267852) | 0.000866 (0.55408) | -1.1e-05 (0.716039) |
| | 1.478548 (<0.0001) | -0.11785 (<0.0001) | 0.002737 (<0.0001) | -0.060341 (0.003639) | 0.0045 (0.00194) | -9.1e-05 (0.003019) |
| | 1.382281 (<0.0001) | -0.108553 (<0.0001) | 0.002463 (<0.0001) | -0.043568 (0.036158) | 0.003166 (0.029088) | -6.3e-05 (0.037765) |
| | 1.494474 (<0.0001) | -0.120808 (<0.0001) | 0.002887 (<0.0001) | -0.016763 (0.423466) | 0.00079 (0.588707) | -1.3e-05 (0.68002) |

| Method | $Age$ ($p$) | $Age^2$ ($p$) | $Age^3$ ($p$) | $Time$ ($p$) | $Time^2$ ($p$) | $Time^3$ ($p$) |
|---|---|---|---|---|---|---|
| | 1.470761 (<0.0001) | -0.118528 (<0.0001) | 0.002815 (<0.0001) | -0.018833 (0.371642) | 0.001263 (0.390297) | -2.8e-05 (0.365204) |
| | 1.458587 (<0.0001) | -0.117286 (<0.0001) | 0.002782 (<0.0001) | -0.013594 (0.509744) | 0.000526 (0.716379) | -1.4e-05 (0.640502) |
| | 1.441864 (<0.0001) | -0.117848 (<0.0001) | 0.002818 (<0.0001) | -0.011221 (0.586412) | 0.000199 (0.889935) | 1.2e-05 (0.6951) |
| | 1.413548 (<0.0001) | -0.113437 (<0.0001) | 0.002682 (<0.0001) | 0.013876 (0.49674) | -0.001498 (0.296447) | 2.6e-05 (0.394443) |
| | 1.346716 (<0.0001) | -0.106522 (<0.0001) | 0.002469 (<0.0001) | 0.022395 (0.280621) | -0.001447 (0.320242) | 2.1e-05 (0.499586) |
| | 1.345751 (<0.0001) | -0.103189 (<0.0001) | 0.002278 (<0.0001) | -0.002915 (0.887453) | -0.000768 (0.592851) | 2.6e-05 (0.391435) |
| | 1.368118 (<0.0001) | -0.107463 (<0.0001) | 0.002459 (<0.0001) | -0.005478 (0.790552) | 3.3e-05 (0.981523) | 1e-06 (0.976322) |
| | 1.480253 (<0.0001) | -0.118944 (<0.0001) | 0.002794 (<0.0001) | -0.010069 (0.623167) | 0.000196 (0.891376) | 6e-06 (0.830876) |
| | 1.326192 (<0.0001) | -0.102482 (<0.0001) | 0.002287 (<0.0001) | -0.004953 (0.808166) | -0.000245 (0.864172) | 9e-06 (0.775369) |
| | 1.351115 (<0.0001) | -0.10755 (<0.0001) | 0.002507 (<0.0001) | 0.013192 (0.517679) | -0.001217 (0.393365) | 2.5e-05 (0.399862) |
| | 1.483649 (<0.0001) | -0.121139 (<0.0001) | 0.002912 (<0.0001) | 0.012019 (0.560765) | -0.000902 (0.533781) | 2.1e-05 (0.486449) |
| | 1.446879 (<0.0001) | -0.118278 (<0.0001) | 0.002837 (<0.0001) | -0.012258 (0.556064) | 0.001572 (0.280706) | -3.6e-05 (0.237239) |
| | 1.527557 (<0.0001) | -0.123504 (<0.0001) | 0.002962 (<0.0001) | -0.042967 (0.038767) | 0.00265 (0.067918) | -5.2e-05 (0.084573) |
| | 1.43545 (<0.0001) | -0.112875 (<0.0001) | 0.002586 (<0.0001) | -0.034076 (0.097255) | 0.002055 (0.153274) | -3.9e-05 (0.197503) |
| | 1.303301 (<0.0001) | -0.100605 (<0.0001) | 0.002249 (<0.0001) | 0.00865 (0.673972) | -0.001382 (0.335993) | 3e-05 (0.319191) |
| | 1.405868 (<0.0001) | -0.108531 (<0.0001) | 0.0024 (<0.0001) | -0.031472 (0.132698) | 0.00254 (0.081823) | -5.3e-05 (0.081686) |
| | 1.399375 (<0.0001) | -0.110676 (<0.0001) | 0.002564 (<0.0001) | -0.01149 (0.581785) | 0.000313 (0.830542) | 1e-06 (0.973605) |
| | 1.281933 (<0.0001) | -0.103361 (<0.0001) | 0.002438 (<0.0001) | 0.020085 (0.326038) | -0.000782 (0.585134) | 8e-06 (0.799232) |
| | 1.349634 (<0.0001) | -0.10585 (<0.0001) | 0.002403 (<0.0001) | -0.004155 (0.838867) | 0.000322 (0.821998) | -4e-06 (0.904015) |
| | 1.364358 (<0.0001) | -0.106072 (<0.0001) | 0.002388 (<0.0001) | 0.008029 (0.699222) | -0.000873 (0.546937) | 1e-05 (0.739627) |
| | 1.443567 (<0.0001) | -0.115641 (<0.0001) | 0.002706 (<0.0001) | -0.012288 (0.554651) | 0.001244 (0.39238) | -3.2e-05 (0.289797) |
| | 1.454254 (<0.0001) | -0.11577 (<0.0001) | 0.002693 (<0.0001) | -0.001591 (0.939025) | -0.000473 (0.744761) | 1e-05 (0.739129) |
| | 1.444097 (<0.0001) | -0.114522 (<0.0001) | 0.002655 (<0.0001) | 0.031836 (0.117793) | -0.00284 (0.048347) | 6e-05 (0.050229) |
| | 1.442224 (<0.0001) | -0.114217 (<0.0001) | 0.00264 (<0.0001) | 0.000895 (0.966115) | -0.000541 (0.712784) | 1e-05 (0.747102) |
| | 1.490825 (<0.0001) | -0.119925 (<0.0001) | 0.002843 (<0.0001) | 0.031324 (0.126492) | -0.002277 (0.113367) | 4.3e-05 (0.159138) |
| | 1.324253 (<0.0001) | -0.100786 (<0.0001) | 0.002203 (<0.0001) | -0.020895 (0.314023) | 0.001074 (0.460326) | -2.6e-05 (0.403222) |
| | 1.426621 (<0.0001) | -0.111815 (<0.0001) | 0.002569 (<0.0001) | -0.011499 (0.584375) | 0.000481 (0.743915) | -1.6e-05 (0.593483) |
| | 1.414004 (<0.0001) | -0.11348 (<0.0001) | 0.002679 (<0.0001) | 0.003697 (0.85685) | -0.000937 (0.512887) | 2.8e-05 (0.356533) |
| | 1.43872 (<0.0001) | -0.116193 (<0.0001) | 0.002741 (<0.0001) | 0.024766 (0.224126) | -0.001858 (0.194665) | 3.9e-05 (0.19801) |
| | 1.298988 (<0.0001) | -0.098369 (<0.0001) | 0.002123 (<0.0001) | 0.008495 (0.677172) | -0.000362 (0.800355) | 4e-06 (0.895529) |
| | 1.387075 (<0.0001) | -0.110072 (<0.0001) | 0.002567 (<0.0001) | -0.016088 (0.435804) | 0.001107 (0.442614) | -2.5e-05 (0.400936) |
| | 1.509474 (<0.0001) | -0.12257 (<0.0001) | 0.002931 (<0.0001) | -0.006449 (0.752771) | 0.001017 (0.478511) | -2.7e-05 (0.369954) |
| | 1.470529 (<0.0001) | -0.118254 (<0.0001) | 0.002779 (<0.0001) | -0.036158 (0.077618) | 0.001852 (0.197812) | -2.5e-05 (0.405801) |
| | 1.573805 (<0.0001) | -0.128209 (<0.0001) | 0.003086 (<0.0001) | -0.010065 (0.622273) | 0.000445 (0.755962) | -1e-06 (0.970441) |
| | 1.406657 (<0.0001) | -0.112822 (<0.0001) | 0.002651 (<0.0001) | 0.01043 (0.616199) | -0.000788 (0.5885) | 1.6e-05 (0.599419) |
| | 1.370864 (<0.0001) | -0.107385 (<0.0001) | 0.002408 (<0.0001) | 0.005866 (0.776976) | 0.00036 (0.802976) | -1.3e-05 (0.657442) |
| | 1.523548 (<0.0001) | -0.126305 (<0.0001) | 0.00308 (<0.0001) | 0.001538 (0.940116) | 0.000577 (0.687226) | -1.7e-05 (0.565294) |
| | 1.378437 (<0.0001) | -0.108065 (<0.0001) | 0.002455 (<0.0001) | -0.034645 (0.097555) | 0.002258 (0.121906) | -4.5e-05 (0.138035) |
| | 1.544045 (<0.0001) | -0.125286 (<0.0001) | 0.002976 (<0.0001) | -0.004757 (0.818638) | 0.000608 (0.675014) | -1.5e-05 (0.633871) |
| | 1.503052 (<0.0001) | -0.120792 (<0.0001) | 0.002857 (<0.0001) | -0.042589 (0.040859) | 0.002571 (0.076724) | -4.8e-05 (0.11748) |
| | 1.390467 (<0.0001) | -0.110119 (<0.0001) | 0.002533 (<0.0001) | -0.002266 (0.912069) | -0.000231 (0.872262) | 9e-06 (0.768442) |
| | 1.414249 (<0.0001) | -0.112108 (<0.0001) | 0.00262 (<0.0001) | -0.003567 (0.861562) | -5.4e-05 (0.970161) | -1e-05 (0.750992) |
| | 1.469364 (<0.0001) | -0.117569 (<0.0001) | 0.002751 (<0.0001) | -0.008028 (0.694807) | 0.000363 (0.800273) | -1e-05 (0.737872) |

| Method | $Age$ ($p$) | $Age^2$ ($p$) | $Age^3$ ($p$) | $Time$ ($p$) | $Time^2$ ($p$) | $Time^3$ ($p$) |
|---|---|---|---|---|---|---|
| | 1.299703 (<0.0001) | -0.098335 (<0.0001) | 0.002127 (<0.0001) | -0.009952 (0.628129) | 0.000137 (0.924038) | -5e-06 (0.873261) |
| | 1.410711 (<0.0001) | -0.113418 (<0.0001) | 0.002681 (<0.0001) | 0.007177 (0.72768) | -0.000763 (0.597231) | 1.6e-05 (0.609111) |
| | 1.338628 (<0.0001) | -0.104918 (<0.0001) | 0.002371 (<0.0001) | -0.01837 (0.37159) | 0.00088 (0.541495) | -1.1e-05 (0.708367) |
| | 1.388275 (<0.0001) | -0.110323 (<0.0001) | 0.00257 (<0.0001) | -0.004447 (0.828335) | -0.000603 (0.675696) | 2.2e-05 (0.471391) |
| | 1.392872 (<0.0001) | -0.11121 (<0.0001) | 0.002604 (<0.0001) | 0.022404 (0.280169) | -0.002197 (0.129836) | 4.9e-05 (0.111219) |
| | 1.42927 (<0.0001) | -0.111038 (<0.0001) | 0.002509 (<0.0001) | -0.016409 (0.428503) | 0.000273 (0.849898) | -2e-06 (0.944615) |
| | 1.386567 (<0.0001) | -0.109781 (<0.0001) | 0.002538 (<0.0001) | -0.000783 (0.969331) | -0.000255 (0.858578) | 1e-06 (0.968916) |
| | 1.35645 (<0.0001) | -0.109237 (<0.0001) | 0.00257 (<0.0001) | 0.001233 (0.952657) | 0.000754 (0.60269) | -2.3e-05 (0.449388) |
| | 1.425244 (<0.0001) | -0.111522 (<0.0001) | 0.002513 (<0.0001) | -0.022883 (0.270143) | 0.000763 (0.598114) | -8e-06 (0.798353) |
| | 1.459754 (<0.0001) | -0.117174 (<0.0001) | 0.002752 (<0.0001) | -0.009631 (0.645646) | 0.001346 (0.358105) | -3.6e-05 (0.244771) |
| | 1.445619 (<0.0001) | -0.116337 (<0.0001) | 0.002743 (<0.0001) | 0.016156 (0.441381) | -0.001039 (0.477864) | 2e-05 (0.52013) |
| | 1.380316 (<0.0001) | -0.108038 (<0.0001) | 0.002443 (<0.0001) | -0.01311 (0.527251) | 0.000851 (0.557448) | -1.8e-05 (0.559665) |
| | 1.44928 (<0.0001) | -0.116439 (<0.0001) | 0.00274 (<0.0001) | -0.005309 (0.800387) | 0.001204 (0.41241) | -3.8e-05 (0.219339) |
| | 1.374484 (<0.0001) | -0.108541 (<0.0001) | 0.002489 (<0.0001) | -0.015462 (0.448542) | 0.001058 (0.458205) | -2.5e-05 (0.408646) |
| | 1.48552 (<0.0001) | -0.119793 (<0.0001) | 0.002831 (<0.0001) | 0.012325 (0.548489) | -0.0006 (0.675096) | 2e-06 (0.943282) |
| | 1.436821 (<0.0001) | -0.114275 (<0.0001) | 0.002644 (<0.0001) | 0.004479 (0.82618) | -0.000806 (0.573507) | 1.6e-05 (0.589287) |
| | 1.407821 (<0.0001) | -0.109305 (<0.0001) | 0.00247 (<0.0001) | -0.009089 (0.661229) | -0.00079 (0.585617) | 2.7e-05 (0.371808) |
| | 1.197875 (<0.0001) | -0.087233 (<0.0001) | 0.001873 (<0.0001) | -0.196814 (<0.0001) | 0.009335 (<0.0001) | -0.000166 (<0.0001) |
| | 1.140093 (<0.0001) | -0.080435 (<0.0001) | 0.001626 (<0.0001) | -0.159657 (<0.0001) | 0.006642 (<0.0001) | -0.00011 (0.000538) |
| | 1.142855 (<0.0001) | -0.080905 (<0.0001) | 0.001656 (<0.0001) | -0.161226 (<0.0001) | 0.005816 (<0.0001) | -8.3e-05 (0.008958) |
| | 1.310469 (<0.0001) | -0.096164 (<0.0001) | 0.002084 (<0.0001) | -0.16336 (<0.0001) | 0.006711 (<0.0001) | -0.000111 (0.000556) |
| | 1.228712 (<0.0001) | -0.087582 (<0.0001) | 0.00182 (<0.0001) | -0.17659 (<0.0001) | 0.007702 (<0.0001) | -0.000132 (<0.0001) |
| | 1.299052 (<0.0001) | -0.097819 (<0.0001) | 0.002179 (<0.0001) | -0.158307 (<0.0001) | 0.006751 (<0.0001) | -0.000118 (0.000212) |
| | 1.223881 (<0.0001) | -0.088469 (<0.0001) | 0.001873 (<0.0001) | -0.174889 (<0.0001) | 0.007812 (<0.0001) | -0.000137 (<0.0001) |
| | 1.314663 (<0.0001) | -0.097038 (<0.0001) | 0.002112 (<0.0001) | -0.2144 (<0.0001) | 0.010245 (<0.0001) | -0.00018 (<0.0001) |
| | 1.342093 (<0.0001) | -0.098732 (<0.0001) | 0.002132 (<0.0001) | -0.179686 (<0.0001) | 0.007656 (<0.0001) | -0.000127 (<0.0001) |
| | 1.246543 (<0.0001) | -0.08966 (<0.0001) | 0.001877 (<0.0001) | -0.191126 (<0.0001) | 0.007977 (<0.0001) | -0.000125 (<0.0001) |
| | 1.186373 (<0.0001) | -0.086792 (<0.0001) | 0.001883 (<0.0001) | -0.160864 (<0.0001) | 0.006351 (<0.0001) | -9.6e-05 (0.002788) |
| | 1.222457 (<0.0001) | -0.08976 (<0.0001) | 0.001926 (<0.0001) | -0.155478 (<0.0001) | 0.006767 (<0.0001) | -0.00012 (0.000189) |
| | 1.283121 (<0.0001) | -0.095167 (<0.0001) | 0.002105 (<0.0001) | -0.194908 (<0.0001) | 0.008616 (<0.0001) | -0.000147 (<0.0001) |
| | 1.294416 (<0.0001) | -0.096725 (<0.0001) | 0.002139 (<0.0001) | -0.159968 (<0.0001) | 0.006477 (<0.0001) | -0.000103 (0.001378) |
| | 1.264627 (<0.0001) | -0.092908 (<0.0001) | 0.002007 (<0.0001) | -0.205591 (<0.0001) | 0.009556 (<0.0001) | -0.000164 (<0.0001) |
| | 1.211946 (<0.0001) | -0.087647 (<0.0001) | 0.00186 (<0.0001) | -0.166185 (<0.0001) | 0.006521 (<0.0001) | -9.5e-05 (0.002908) |
| VAMBN-MT (ii) 10,000 | 1.137246 (<0.0001) | -0.078739 (<0.0001) | 0.001557 (<0.0001) | -0.185759 (<0.0001) | 0.008095 (<0.0001) | -0.000138 (<0.0001) |
| | 1.336356 (<0.0001) | -0.098988 (<0.0001) | 0.002161 (<0.0001) | -0.191472 (<0.0001) | 0.008663 (<0.0001) | -0.000148 (<0.0001) |
| | 1.315905 (<0.0001) | -0.095873 (<0.0001) | 0.002035 (<0.0001) | -0.19204 (<0.0001) | 0.008293 (<0.0001) | -0.000138 (<0.0001) |
| | 1.255946 (<0.0001) | -0.092612 (<0.0001) | 0.00203 (<0.0001) | -0.174545 (<0.0001) | 0.007035 (<0.0001) | -0.000113 (0.000464) |
| | 1.418519 (<0.0001) | -0.10828 (<0.0001) | 0.002466 (<0.0001) | -0.173419 (<0.0001) | 0.006532 (<0.0001) | -9.6e-05 (0.00202) |
| | 1.37659 (<0.0001) | -0.106386 (<0.0001) | 0.002482 (<0.0001) | -0.189723 (<0.0001) | 0.008941 (<0.0001) | -0.000161 (<0.0001) |
| | 1.265672 (<0.0001) | -0.093292 (<0.0001) | 0.002011 (<0.0001) | -0.156296 (<0.0001) | 0.006241 (<0.0001) | -9.6e-05 (0.002459) |
| | 1.276042 (<0.0001) | -0.094103 (<0.0001) | 0.002041 (<0.0001) | -0.14568 (<0.0001) | 0.005661 (0.000161) | -9.1e-05 (0.004708) |
| | 1.139085 (<0.0001) | -0.080644 (<0.0001) | 0.001658 (<0.0001) | -0.171934 (<0.0001) | 0.007919 (<0.0001) | -0.000146 (<0.0001) |
| | 1.231197 (<0.0001) | -0.088917 (<0.0001) | 0.001884 (<0.0001) | -0.187278 (<0.0001) | 0.008428 (<0.0001) | -0.000146 (<0.0001) |

| Method | $Age$ ($p$) | $Age^2$ ($p$) | $Age^3$ ($p$) | $Time$ ($p$) | $Time^2$ ($p$) | $Time^3$ ($p$) |
|---|---|---|---|---|---|---|
| | 1.266312 (<0.0001) | -0.094724 (<0.0001) | 0.0021 (<0.0001) | -0.19022 (<0.0001) | 0.008875 (<0.0001) | -0.000158 (<0.0001) |
| | 1.268357 (<0.0001) | -0.093894 (<0.0001) | 0.002051 (<0.0001) | -0.211777 (<0.0001) | 0.010461 (<0.0001) | -0.000189 (<0.0001) |
| | 1.29376 (<0.0001) | -0.095616 (<0.0001) | 0.002093 (<0.0001) | -0.194139 (<0.0001) | 0.008349 (<0.0001) | -0.000132 (<0.0001) |
| | 1.254384 (<0.0001) | -0.091245 (<0.0001) | 0.001955 (<0.0001) | -0.1931 (<0.0001) | 0.008603 (<0.0001) | -0.000143 (<0.0001) |
| | 1.420929 (<0.0001) | -0.106224 (<0.0001) | 0.002346 (<0.0001) | -0.199873 (<0.0001) | 0.00934 (<0.0001) | -0.00016 (<0.0001) |
| | 1.224548 (<0.0001) | -0.087484 (<0.0001) | 0.001827 (<0.0001) | -0.183936 (<0.0001) | 0.008275 (<0.0001) | -0.000143 (<0.0001) |
| | 1.289497 (<0.0001) | -0.0967 (<0.0001) | 0.002133 (<0.0001) | -0.160829 (<0.0001) | 0.00649 (<0.0001) | -0.000101 (0.001291) |
| | 1.271421 (<0.0001) | -0.093913 (<0.0001) | 0.002055 (<0.0001) | -0.18703 (<0.0001) | 0.008627 (<0.0001) | -0.000155 (<0.0001) |
| | 1.311954 (<0.0001) | -0.098954 (<0.0001) | 0.002231 (<0.0001) | -0.165116 (<0.0001) | 0.006875 (<0.0001) | -0.000114 (0.000403) |
| | 1.317976 (<0.0001) | -0.098538 (<0.0001) | 0.002171 (<0.0001) | -0.175963 (<0.0001) | 0.00782 (<0.0001) | -0.00013 (<0.0001) |
| | 1.271006 (<0.0001) | -0.093908 (<0.0001) | 0.002041 (<0.0001) | -0.147005 (<0.0001) | 0.005296 (0.000326) | -8e-05 (0.010291) |
| | 1.288979 (<0.0001) | -0.091541 (<0.0001) | 0.001884 (<0.0001) | -0.184931 (<0.0001) | 0.008111 (<0.0001) | -0.000135 (<0.0001) |
| | 1.415764 (<0.0001) | -0.106503 (<0.0001) | 0.002372 (<0.0001) | -0.198219 (<0.0001) | 0.008766 (<0.0001) | -0.000146 (<0.0001) |
| | 1.213079 (<0.0001) | -0.087217 (<0.0001) | 0.001839 (<0.0001) | -0.189393 (<0.0001) | 0.007878 (<0.0001) | -0.000127 (<0.0001) |
| | 1.142052 (<0.0001) | -0.080149 (<0.0001) | 0.001605 (<0.0001) | -0.15201 (<0.0001) | 0.006751 (<0.0001) | -0.000123 (<0.0001) |
| | 1.402261 (<0.0001) | -0.107284 (<0.0001) | 0.00246 (<0.0001) | -0.17991 (<0.0001) | 0.007561 (<0.0001) | -0.000121 (0.000167) |
| | 1.290111 (<0.0001) | -0.094875 (<0.0001) | 0.00205 (<0.0001) | -0.200172 (<0.0001) | 0.009331 (<0.0001) | -0.000164 (<0.0001) |
| | 1.357088 (<0.0001) | -0.103223 (<0.0001) | 0.002343 (<0.0001) | -0.206863 (<0.0001) | 0.009772 (<0.0001) | -0.000169 (<0.0001) |
| | 1.106836 (<0.0001) | -0.078241 (<0.0001) | 0.001594 (<0.0001) | -0.192259 (<0.0001) | 0.008746 (<0.0001) | -0.00015 (<0.0001) |
| | 1.303812 (<0.0001) | -0.098496 (<0.0001) | 0.002214 (<0.0001) | -0.183922 (<0.0001) | 0.008632 (<0.0001) | -0.000156 (<0.0001) |
| | 1.201231 (<0.0001) | -0.085696 (<0.0001) | 0.001765 (<0.0001) | -0.193588 (<0.0001) | 0.008456 (<0.0001) | -0.000138 (<0.0001) |
| | 1.190787 (<0.0001) | -0.087772 (<0.0001) | 0.0019 (<0.0001) | -0.183968 (<0.0001) | 0.007817 (<0.0001) | -0.000122 (0.000119) |
| | 1.278096 (<0.0001) | -0.094334 (<0.0001) | 0.002053 (<0.0001) | -0.195062 (<0.0001) | 0.00917 (<0.0001) | -0.000163 (<0.0001) |
| | 1.272487 (<0.0001) | -0.094134 (<0.0001) | 0.002052 (<0.0001) | -0.177653 (<0.0001) | 0.007301 (<0.0001) | -0.000114 (0.000268) |
| | 1.265323 (<0.0001) | -0.094337 (<0.0001) | 0.002081 (<0.0001) | -0.173451 (<0.0001) | 0.007676 (<0.0001) | -0.000132 (<0.0001) |
| | 1.280096 (<0.0001) | -0.09472 (<0.0001) | 0.002076 (<0.0001) | -0.20432 (<0.0001) | 0.009496 (<0.0001) | -0.000164 (<0.0001) |
| | 1.324767 (<0.0001) | -0.096675 (<0.0001) | 0.002079 (<0.0001) | -0.180672 (<0.0001) | 0.007714 (<0.0001) | -0.00013 (<0.0001) |
| | 1.271809 (<0.0001) | -0.094074 (<0.0001) | 0.002042 (<0.0001) | -0.158978 (<0.0001) | 0.006643 (<0.0001) | -0.000112 (0.000493) |
| | 1.415406 (<0.0001) | -0.108458 (<0.0001) | 0.002491 (<0.0001) | -0.17482 (<0.0001) | 0.00677 (<0.0001) | -9.8e-05 (0.002064) |
| | 1.213286 (<0.0001) | -0.087185 (<0.0001) | 0.001808 (<0.0001) | -0.15224 (<0.0001) | 0.00592 (<0.0001) | -9.5e-05 (0.002324) |
| | 1.315186 (<0.0001) | -0.096216 (<0.0001) | 0.002072 (<0.0001) | -0.238751 (<0.0001) | 0.011864 (<0.0001) | -0.000209 (<0.0001) |
| | 1.192898 (<0.0001) | -0.083992 (<0.0001) | 0.00169 (<0.0001) | -0.185915 (<0.0001) | 0.008143 (<0.0001) | -0.000135 (<0.0001) |
| | 1.200757 (<0.0001) | -0.086666 (<0.0001) | 0.001829 (<0.0001) | -0.169705 (<0.0001) | 0.007251 (<0.0001) | -0.000126 (<0.0001) |
| | 1.298997 (<0.0001) | -0.097433 (<0.0001) | 0.002151 (<0.0001) | -0.189486 (<0.0001) | 0.008285 (<0.0001) | -0.000137 (<0.0001) |
| | 1.353886 (<0.0001) | -0.102239 (<0.0001) | 0.002293 (<0.0001) | -0.186484 (<0.0001) | 0.008792 (<0.0001) | -0.000156 (<0.0001) |
| | 1.351991 (<0.0001) | -0.103287 (<0.0001) | 0.002339 (<0.0001) | -0.168583 (<0.0001) | 0.007063 (<0.0001) | -0.000116 (0.000265) |
| | 1.236619 (<0.0001) | -0.090211 (<0.0001) | 0.001924 (<0.0001) | -0.194118 (<0.0001) | 0.008972 (<0.0001) | -0.000154 (<0.0001) |
| | 1.201102 (<0.0001) | -0.086973 (<0.0001) | 0.001841 (<0.0001) | -0.193079 (<0.0001) | 0.008736 (<0.0001) | -0.000148 (<0.0001) |
| | 1.319192 (<0.0001) | -0.098446 (<0.0001) | 0.002186 (<0.0001) | -0.169736 (<0.0001) | 0.007168 (<0.0001) | -0.000121 (0.000162) |
| | 1.188878 (<0.0001) | -0.084317 (<0.0001) | 0.001699 (<0.0001) | -0.194531 (<0.0001) | 0.00945 (<0.0001) | -0.000169 (<0.0001) |
| | 1.187222 (<0.0001) | -0.085435 (<0.0001) | 0.001774 (<0.0001) | -0.166812 (<0.0001) | 0.007237 (<0.0001) | -0.000124 (<0.0001) |
| | 1.386118 (<0.0001) | -0.104169 (<0.0001) | 0.00231 (<0.0001) | -0.162555 (<0.0001) | 0.006191 (<0.0001) | -9.3e-05 (0.003633) |
| | 1.304145 (<0.0001) | -0.097004 (<0.0001) | 0.002137 (<0.0001) | -0.188486 (<0.0001) | 0.008582 (<0.0001) | -0.00015 (<0.0001) |

| Method | $Age$ ($p$) | $Age^2$ ($p$) | $Age^3$ ($p$) | $Time$ ($p$) | $Time^2$ ($p$) | $Time^3$ ($p$) |
|---|---|---|---|---|---|---|
| | 1.229122 (<0.0001) | -0.087063 (<0.0001) | 0.001764 (<0.0001) | -0.179439 (<0.0001) | 0.007615 (<0.0001) | -0.000125 (<0.0001) |
| | 1.288569 (<0.0001) | -0.096806 (<0.0001) | 0.002168 (<0.0001) | -0.16137 (<0.0001) | 0.006719 (<0.0001) | -0.000114 (0.000385) |
| | 1.138803 (<0.0001) | -0.077777 (<0.0001) | 0.001482 (<0.0001) | -0.182281 (<0.0001) | 0.007682 (<0.0001) | -0.000128 (<0.0001) |
| | 1.355529 (<0.0001) | -0.102935 (<0.0001) | 0.00233 (<0.0001) | -0.195753 (<0.0001) | 0.009209 (<0.0001) | -0.000162 (<0.0001) |
| | 1.286729 (<0.0001) | -0.095252 (<0.0001) | 0.002084 (<0.0001) | -0.173796 (<0.0001) | 0.00748 (<0.0001) | -0.000122 (0.000128) |
| | 1.284461 (<0.0001) | -0.094719 (<0.0001) | 0.002058 (<0.0001) | -0.154055 (<0.0001) | 0.006413 (<0.0001) | -0.000106 (0.001001) |
| | 1.356769 (<0.0001) | -0.100482 (<0.0001) | 0.002188 (<0.0001) | -0.176943 (<0.0001) | 0.007307 (<0.0001) | -0.000116 (0.000256) |
| | 1.343944 (<0.0001) | -0.101608 (<0.0001) | 0.002291 (<0.0001) | -0.191069 (<0.0001) | 0.008108 (<0.0001) | -0.000134 (<0.0001) |
| | 1.181608 (<0.0001) | -0.084683 (<0.0001) | 0.001756 (<0.0001) | -0.167864 (<0.0001) | 0.006736 (<0.0001) | -0.000107 (0.000866) |
| | 1.279754 (<0.0001) | -0.09312 (<0.0001) | 0.001983 (<0.0001) | -0.200026 (<0.0001) | 0.009071 (<0.0001) | -0.000156 (<0.0001) |
| | 1.336283 (<0.0001) | -0.100883 (<0.0001) | 0.002282 (<0.0001) | -0.159467 (<0.0001) | 0.005615 (0.000171) | -7.9e-05 (0.012905) |
| | 1.231404 (<0.0001) | -0.089336 (<0.0001) | 0.0019 (<0.0001) | -0.234684 (<0.0001) | 0.012389 (<0.0001) | -0.000233 (<0.0001) |
| | 1.259017 (<0.0001) | -0.090834 (<0.0001) | 0.001906 (<0.0001) | -0.16242 (<0.0001) | 0.006533 (<0.0001) | -0.000107 (0.000706) |
| | 1.286087 (<0.0001) | -0.095839 (<0.0001) | 0.002116 (<0.0001) | -0.171082 (<0.0001) | 0.007834 (<0.0001) | -0.00014 (<0.0001) |
| | 1.243217 (<0.0001) | -0.090548 (<0.0001) | 0.001924 (<0.0001) | -0.187799 (<0.0001) | 0.008103 (<0.0001) | -0.000132 (<0.0001) |
| | 1.464282 (<0.0001) | -0.114637 (<0.0001) | 0.002709 (<0.0001) | -0.180623 (<0.0001) | 0.008349 (<0.0001) | -0.000147 (<0.0001) |
| | 1.275468 (<0.0001) | -0.096105 (<0.0001) | 0.002154 (<0.0001) | -0.1627 (<0.0001) | 0.006911 (<0.0001) | -0.000116 (0.00027) |
| | 1.316188 (<0.0001) | -0.099817 (<0.0001) | 0.002253 (<0.0001) | -0.177192 (<0.0001) | 0.007406 (<0.0001) | -0.000121 (0.000144) |
| | 1.26229 (<0.0001) | -0.091236 (<0.0001) | 0.001942 (<0.0001) | -0.169022 (<0.0001) | 0.007051 (<0.0001) | -0.000119 (0.000196) |
| | 1.205653 (<0.0001) | -0.086532 (<0.0001) | 0.001799 (<0.0001) | -0.211661 (<0.0001) | 0.009996 (<0.0001) | -0.000171 (<0.0001) |
| | 1.235243 (<0.0001) | -0.090141 (<0.0001) | 0.001909 (<0.0001) | -0.165754 (<0.0001) | 0.006425 (<0.0001) | -0.0001 (0.001471) |
| | 1.342381 (<0.0001) | -0.101417 (<0.0001) | 0.002301 (<0.0001) | -0.211247 (<0.0001) | 0.009795 (<0.0001) | -0.000165 (<0.0001) |
| | 1.291467 (<0.0001) | -0.094846 (<0.0001) | 0.002044 (<0.0001) | -0.19375 (<0.0001) | 0.008863 (<0.0001) | -0.00015 (<0.0001) |
| | 1.439101 (<0.0001) | -0.10984 (<0.0001) | 0.002512 (<0.0001) | -0.177249 (<0.0001) | 0.007251 (<0.0001) | -0.000116 (0.000272) |
| | 1.306405 (<0.0001) | -0.098516 (<0.0001) | 0.002212 (<0.0001) | -0.131887 (<0.0001) | 0.004333 (0.004139) | -5.7e-05 (0.075286) |
| | 1.196009 (<0.0001) | -0.085871 (<0.0001) | 0.001801 (<0.0001) | -0.166548 (<0.0001) | 0.00645 (<0.0001) | -9.9e-05 (0.002066) |
| | 1.271511 (<0.0001) | -0.094543 (<0.0001) | 0.002093 (<0.0001) | -0.156238 (<0.0001) | 0.006067 (<0.0001) | -9.5e-05 (0.003111) |
| | 1.204346 (<0.0001) | -0.0888 (<0.0001) | 0.001933 (<0.0001) | -0.162184 (<0.0001) | 0.00627 (<0.0001) | -9.9e-05 (0.001874) |
| | 1.268871 (<0.0001) | -0.092455 (<0.0001) | 0.001986 (<0.0001) | -0.144969 (<0.0001) | 0.005069 (0.000789) | -6.8e-05 (0.034197) |
| | 1.263455 (<0.0001) | -0.090325 (<0.0001) | 0.001875 (<0.0001) | -0.191276 (<0.0001) | 0.008352 (<0.0001) | -0.000137 (<0.0001) |
| | 1.204791 (<0.0001) | -0.086846 (<0.0001) | 0.001811 (<0.0001) | -0.15736 (<0.0001) | 0.006807 (<0.0001) | -0.000122 (0.000102) |

*Note that the values for the original data slightly deviate from the values determined by Perrar *et al.* [2], as we re-built the polynomial mixed-effects models in R, that were originally coded in SAS.

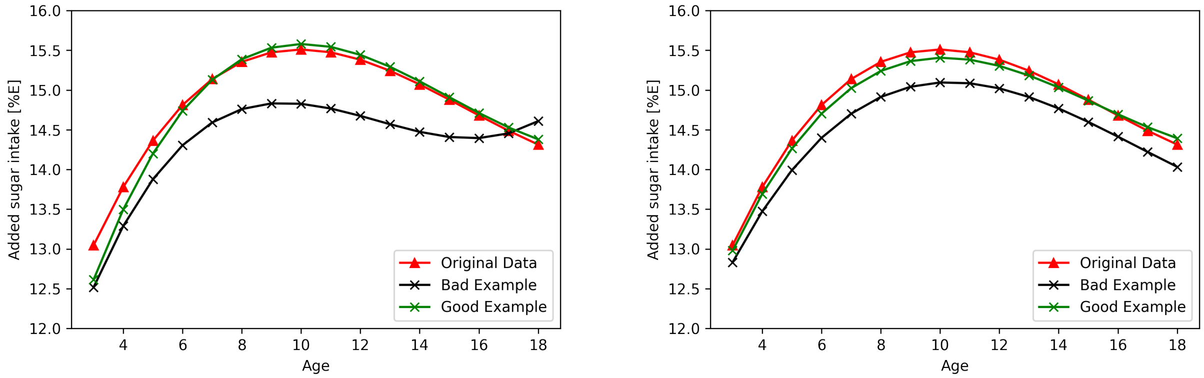


(a) $N = 1,312$ (b) $N = 10,000$

Figure S1: Effects of sample size for the stability of determined age trends. The good and bad examples (shown in green and black, respectively) are manually picked from a set of 100 samples for both sample sizes.

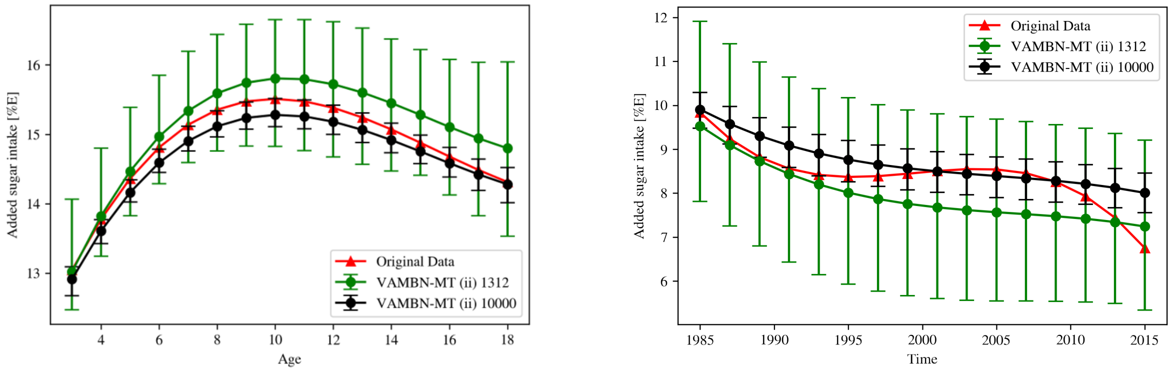


(a) Age trend (b) Time trend

Figure S2: Effects of sample size for age and time trends for added sugar intake predicted by polynomial mixed-effect regression models. Similar to Fig. 8, we show averaged age and time trends of the synthetic datasets in comparison the the real data. Additionally, we show the results for two sample sizes, i.e., 1,312 (green) and 10,000 (black). For the smaller sample size (that corresponds to the original data size), also missingness is inserted.

## Application to a further use case

For a proof of concept of our developed method, we further tested it on the ADNI data, obtained from the *Alzheimer's Disease Neuroimaging Initiative (ADNI)* (adni.loni.usc.edu).

### ADNI data

The ADNI was launched in 2003 as a public-private partnership, led by Principal Investigator Michael W. Weiner, MD. The primary goal of ADNI has been to test whether serial magnetic resonance imaging (MRI), positron emission tomography (PET), other biological markers, and clinical and neuropsychological assessment can be combined to measure the progression of mild cognitive impairment (MCI) and early Alzheimer's disease (AD). Up-to-date information on the available datasets can be found at www.adni-info.org.

The research encompasses a cohort of 417 individuals without cognitive impairment, 106 subjects exhibiting significant memory concerns, 310 subjects with early-stage mild cognitive impairment, and 562 subjects with

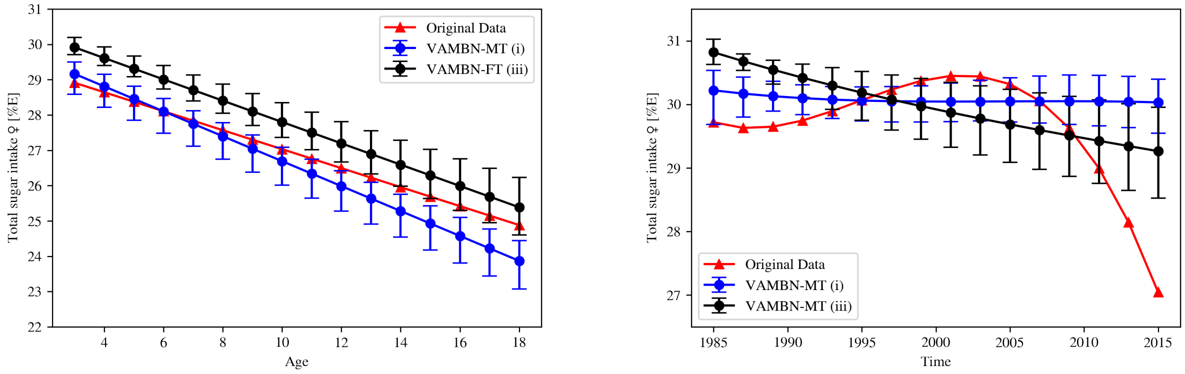


(a) Age trend

(b) Time trend

Figure S3: Age and time trends for the total sugar consumption. Whereas the synthetic data can reproduce the age trend, they fail to reproduce the time trend, independent on the module selection (i.e., whether time and total sugar are learned in one module or not). In the real data, only the age trend shows statistically significant values for all terms (linear, quadratic and cubic). For the time trend, both linear and quadratic show non-significant results.

late-stage mild cognitive impairment. Additionally, 342 subjects were initially diagnosed with Alzheimer's disease (AD) at the study's commencement. For this investigation, longitudinal data from 689 patients were utilized, including those initially diagnosed with AD (n=342) or those who progressed to AD during the study. The ADNI dataset comprises genotype information based on single-nucleotide polymorphisms (SNPs), APOE4 status, cerebrospinal fluid (CSF) biomarkers, volume measurements of seven brain regions, and various clinical and neuropsychological test results. The features measured in ADNI were categorized into brain volumes, cortical brain regions, cognition tests, CSF markers, genotype (SNPs + APOE4 status), demographic features, and baseline diagnosis. Features with more than 50% missing values were generally excluded, resulting in our approach modeling visits at baseline, month 6, month 12, and month 24. For further information on ADNIMERGE dataset and the corresponding pre- and post-processing steps refer to our previous work [5].

## Methods

To further evaluate the proposed algorithm, we compare VAMBN with our extension VAMBN-MT. Therefore, we assess the individual variable distributions based on the JS-divergence, and compare the correlation matrices. Our previous work [5] had shown the significance of the original VAMBN model for synthesising ADNI data. To further highlight the benefit of the MT extension for the current study, we have focused on the longitudinal variable groups, namely Volume (VOL) and Cognitive Test (COGT), from ADNIMERGE captured from four subsequent patient visits at baseline (VIS1), month 6 (VIS6), month 12 (VIS12), and month 24 (VIS24).

Table S5: The Jensen-Shannon (JS) divergences for 56 longitudinal variables from ADNI dataset for synthesised data using the original VAMBN model and the VAMBN-MT model.

| **Variable** | **VAMBN** | **VAMBN-MT** |
|---|---|---|
| COGT_ADAS11_VIS24 | 0.178 | 0.141 |
| COGT_RAVLT.immediate_VIS1 | 0.162 | 0.128 |
| COGT_RAVLT.immediate_VIS6 | 0.164 | 0.149 |
| VOL_Fusiform_VIS12 | 0.108 | 0.083 |
| VOL_MidTemp_VIS12 | 0.116 | 0.117 |
| VOL_MidTemp_VIS1 | 0.099 | 0.089 |
| COGT_RAVLT.learning_VIS24 | 0.484 | 0.458 |
| VOL_Entorhinal_VIS1 | 0.091 | 0.083 |
| VOL_Fusiform_VIS1 | 0.096 | 0.080 |

| **Variable** | **VAMBN** | **VAMBN-MT** |
|---|---|---|
| COGT_RAVLT.learning_VIS12 | 0.489 | 0.495 |
| VOL_Ventricles_VIS12 | 0.112 | 0.118 |
| VOL_Entorhinal_VIS12 | 0.102 | 0.076 |
| VOL_WholeBrain_VIS1 | 0.081 | 0.081 |
| COGT_MMSE_VIS1 | 0.238 | 0.262 |
| COGT_RAVLT.immediate_VIS12 | 0.185 | 0.179 |
| COGT_CDRSB_VIS12 | 0.213 | 0.237 |
| COGT_CDRSB_VIS24 | 0.135 | 0.129 |
| COGT_CDRSB_VIS6 | 0.178 | 0.192 |
| COGT_ADAS13_VIS1 | 0.126 | 0.103 |
| COGT_ADAS13_VIS12 | 0.125 | 0.133 |
| COGT_RAVLT.immediate_VIS24 | 0.174 | 0.241 |
| COGT_FAQ_VIS12 | 0.190 | 0.130 |
| VOL_Hippocampus_VIS6 | 0.070 | 0.096 |
| COGT_MMSE_VIS12 | 0.208 | 0.228 |
| VOL_MidTemp_VIS6 | 0.108 | 0.112 |
| VOL_Entorhinal_VIS6 | 0.110 | 0.107 |
| COGT_FAQ_VIS6 | 0.222 | 0.190 |
| VOL_WholeBrain_VIS24 | 0.128 | 0.106 |
| COGT_RAVLT.forgetting_VIS12 | 0.360 | 0.360 |
| COGT_ADAS13_VIS6 | 0.095 | 0.112 |
| VOL_Hippocampus_VIS12 | 0.098 | 0.094 |
| VOL_ICV_VIS24 | 0.191 | 0.079 |
| COGT_MMSE_VIS6 | 0.137 | 0.140 |
| COGT_CDRSB_VIS1 | 0.183 | 0.163 |
| VOL_ICV_VIS1 | 0.097 | 0.059 |
| VOL_Ventricles_VIS1 | 0.091 | 0.105 |
| VOL_Ventricles_VIS24 | 0.100 | 0.090 |
| VOL_Fusiform_VIS6 | 0.088 | 0.094 |
| VOL_ICV_VIS6 | 0.068 | 0.067 |
| COGT_RAVLT.forgetting_VIS1 | 0.353 | 0.381 |
| COGT_FAQ_VIS1 | 0.213 | 0.147 |
| VOL_Hippocampus_VIS1 | 0.108 | 0.104 |
| COGT_RAVLT.forgetting_VIS24 | 0.320 | 0.326 |
| COGT_RAVLT.forgetting_VIS6 | 0.372 | 0.372 |
| COGT_ADAS13_VIS24 | 0.149 | 0.148 |
| COGT_ADAS11_VIS1 | 0.075 | 0.066 |
| VOL_ICV_VIS12 | 0.115 | 0.071 |
| COGT_ADAS11_VIS6 | 0.124 | 0.103 |
| COGT_FAQ_VIS24 | 0.197 | 0.181 |
| COGT_RAVLT.learning_VIS6 | 0.478 | 0.518 |
| COGT_RAVLT.learning_VIS1 | 0.391 | 0.382 |
| COGT_MMSE_VIS24 | 0.256 | 0.311 |
| VOL_Ventricles_VIS6 | 0.102 | 0.088 |
| VOL_WholeBrain_VIS12 | 0.116 | 0.062 |
| VOL_WholeBrain_VIS6 | 0.099 | 0.090 |
| COGT_ADAS11_VIS12 | 0.107 | 0.102 |
| **Average** | $0.175 \pm 0.107$ | $0.167 \pm 0.115$ |

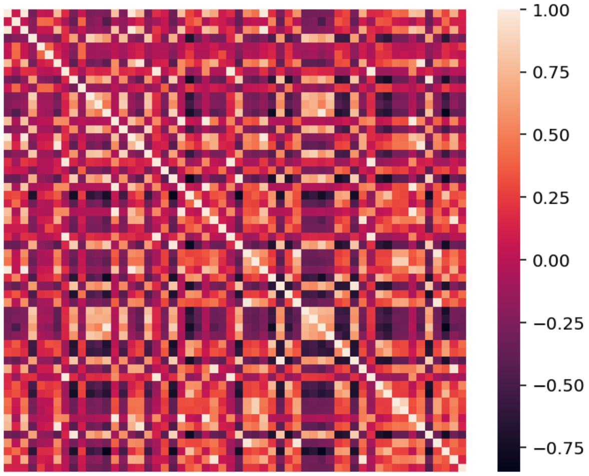


(a) Original Data

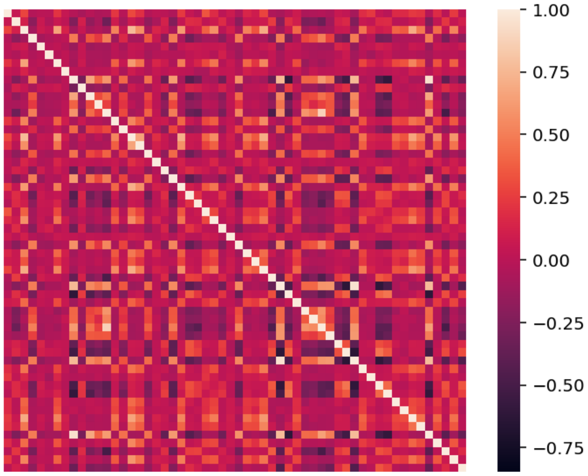


(b) VAMBN ($\epsilon = 0.546$)

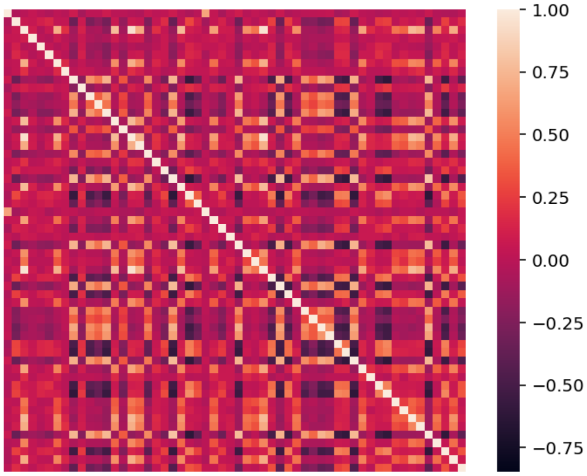


(c) VAMBN-MT ($\epsilon = 0.541$)

Figure S4: Heatmaps of the Pearson correlation matrices of all visits for every variable from ADNI dataset for: the real data (a), the synthetic data produced by VAMBN (b), and the synthetic data produced by VAMBN-MT (c).

# References


[1] Tim J Cole, Mary C Bellizzi, Katherine M Flegal, and William H Dietz. Establishing a standard definition for child overweight and obesity worldwide: international survey. *Bmj*, 320(7244):1240, 2000.

[2] Ines Perrar, Sarah Schmitting, Karen W Della Corte, Anette E Buyken, and Ute Alexy. Age and time trends in sugar intake among children and adolescents: results from the DONALD study. *European journal of nutrition*, 59(3):1043–1054, 2020.

[3] W. N. Schofield. Predicting basal metabolic rate, new standards and review of previous work. *Human nutrition : clinical nutrition*, 1985.

[4] W. Sichert-Hellert, M. Kersting, and G. Schöch. Underreporting of energy intake in 1 to 18 year old german children and adolescents. *Zeitschrift Fur Ernahrungswissenschaft*, 37(3):242–251, 1998.

[5] Meemansa Sood, Akrishta Sahay, Reagon Karki, Mohammad Asif Emon, Henri Vrooman, Martin Hofmann-Apitius, and Holger Fröhlich. Realistic simulation of virtual multi-scale, multi-modal patient trajectories using bayesian networks and sparse auto-encoders. *Scientific Reports*, 10(1):10971, 2020. Number: 1 Publisher: Nature Publishing Group.
